# Pantopaque: Does It Still Migrate With Gravity After 30 Years?

**DOI:** 10.7759/cureus.27566

**Published:** 2022-08-01

**Authors:** Frank Ma, Jamie Jacobs, Linh Bui, Antonio K Liu

**Affiliations:** 1 Internal Medicine, Adventist Health White Memorial, Los Angeles, USA; 2 Radiology, Adventist Health White Memorial, Los Angeles, USA; 3 Neurology, Adventist Health White Memorial, Los Angeles, USA; 4 Neurology, Loma Linda University School of Medicine, Loma Linda, USA

**Keywords:** arachnoiditis, contrast, myelogram, migrate, pantopaque

## Abstract

Pantopaque was an oil-based positive contrast media used in central nervous system imaging before the use of water-soluble contrast agents. It is no longer used due to side effects, including arachnoiditis. Prior studies have indicated that remnants of pantopaque can be seen in modern radiographic imaging, including CT and MRI. With its use obsolete, these remnants have been increasingly mislabeled from “tumor” to “shot gun pellets”. An understanding of this historic modality will usually lead to the correct diagnosis.

## Introduction

Pantopaque, also known as Iogendylate and Myodil (iodophenylundecylic acid), is an oil-based positive contrast media. It was used in myelography, ventriculography, and cisternography from 1944 until 1988, before the broad utilization of computed tomography (CT), magnetic resonance imaging (MRI), and water-soluble contrast agents [1]. Oil-based contrast agents such as pantopaque are known to have potential complications such as arachnoiditis, chronic irritation, and anaphylaxis [2]. It has since been phased out in favor of water-soluble contrast agents. Since its discontinuation, imaging studies with findings suspicious of intradural oil-based contrast have rarely been reported. Although few, there have been documented cases of pantopaque noted on imaging in the spine and intracranially [3-6]. Many of these cases noted pantopaque exposure many decades before presentation, with abnormal imaging noted on CT, MRI, and dental panoramic radiographs. Previous cases have noted the MRI appearance of pantopaque as hyperintense on T1-weighted images and iso- to hypointense on T2-weighted images without gadolinium enhancement [3,5].

We report a case of a 42-year-old woman who had a myelogram performed with pantopaque over 30 years ago after a traumatic motor vehicle accident. During a recent hospitalization, she was incidentally found to have abnormal CT head findings while assessing for a new onset headache. The purpose of this article is not only to remind clinicians of this disappearing phenomenon but also to assess the effects of gravity on hyperdense spots noted on CT imaging intracranially in a patient who likely had pantopaque exposure with a myelogram 35 years ago.

## Case presentation

**Figure 1:**
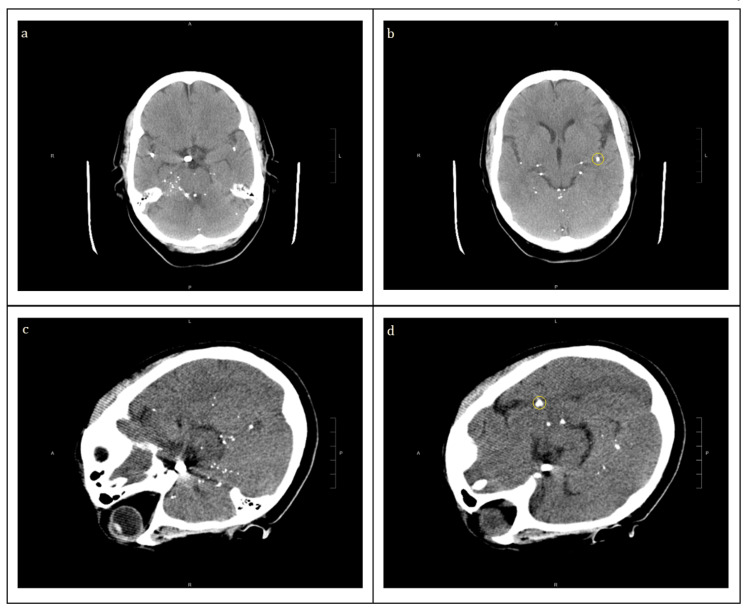
(a, b) CT brain without contrast showing multiple small round hyperdense spots in the sulci of cerebrum and cerebellum, distributing along the meninges, tentorium, and in the internal auditory canals bilaterally. (c, d) Repeat CT brain is performed with the right side of the head down for two-and-half hours, again showing innumerable hyperdense foci appearing similar in distribution; for reference, the yellow circles in (b) and (d) show the same hyperdense spot without migration.

The patient is a 42-year-old woman with a known history of paraplegia secondary to a severe motor vehicle accident at the level of T10 when she was six years old in Guatemala. She has a history of the neurogenic bladder on self-catheterization, frequent urinary tract infections, left-sided kidney stones, and recurrent urinary tract infections. She presented to the hospital emergency department with worsening bilateral flank pain for three days with associated fever. The patient was found to be febrile to 102.4, with leukocytosis and urinalysis with moderate leukocyte esterase, elevated WBC, and RBC count. Over the hospital course, the patient was treated for a urinary tract infection. Additionally, the patient noted new onset headaches before her history of fever, so a head CT was ordered. Multiple hyperdense spots were noted on the CT scan (Figure 1). Neurological examination revealed a coherent and oriented patient. Her cranial nerves II-XII and upper extremities were normal. The lower extremity was noted to be chronically contracted and spastic. The patient had no feeling below T10 and no bowel or bladder control; these findings were the sequelae of the motor vehicle accident. 

Further history was obtained regarding the motor vehicle accident she was in when she was six years old. She recalled having multiple procedures and CT scans of her head and spine that might have included inserting needles in the back and injecting contrast. Since that acute hospitalization and rehabilitation, she has not had any imaging performed on her brain or spine, nor further interventions. The patient and physician had an interesting question: Would these “spots” move with gravity depending on head position? The patient consented to a repeat CT scan after lying flat with her head turned to the right side for two-and-half hours. Interestingly, there was no migration with gravity due to the positioning of the head.

## Discussion

The remnants of pantopaque in this patient’s case, fortunately, did not appear to manifest clinical symptoms. Based on the relative anatomy seen from repeat CT imaging after having the patient lying on the right side of her head for two-and-half hours, there does not seem to be an apparent effect of gravity to displace the location of hyperdense spots (Figure 1).

A PubMed search under the word “pantopaque” yielded a total of 454 entries. It started in 1944 and ended in 2019, with a bell-shaped curve peaking in 1978 (28 papers). It has since steadily decreased with no more than three papers per year since 1996. There were no papers in 2020 and 2021.

Although no longer performed, pneumoencephalography was previously reported to be done for the removal of residual intracranial pantopaque by the introduction of room air into the subarachnoid space [7]. This suggests that the material within the subarachnoid space is displaceable with pressure and positional changes under atmospheric pressure through a spinal needle, although not as much by gravitational force in a closed system [8].

It is still unclear if migration may be a function of time under the effects of gravity. Pantopaque is cleared after it is encysted by CSF, followed by a slow rate of absorption. As such, long-term follow-up with repeat MRI or CT imaging may show decreasing hyperintensity, although this is not known to be reported.

## Conclusions

This serves as a reminder to diagnosticians of the historical use of pantopaque as a contrast agent with evidence of its residual effects that can still be encountered in today’s practice. Although disappearing with time, the occurrence of irregular CT brain findings with multiple hyperdense spots should prompt suspicion and inquiry regarding the history of pantopaque use.
